# Scenario Development as a Basis for Formulating a Research Program on Future Agriculture: A Methodological Approach

**DOI:** 10.1007/s13280-013-0417-3

**Published:** 2013-07-09

**Authors:** Ingrid Öborn, Jan Bengtsson, Fredrik Hedenus, Lotta Rydhmer, Maria Stenström, Katarina Vrede, Charles Westin, Ulf Magnusson

**Affiliations:** 1 Department of Crop Production Ecology, Swedish University of Agricultural Sciences (SLU), Box 7043, Ulls väg 16, 750 07 Uppsala, Sweden; 2 World Agroforestry Centre (ICRAF), P.O. Box 30677-00100, UN Avenue, Nairobi, Kenya; 3 Department of Ecology, Swedish University of Agricultural Sciences, Box 7044, 750 07 Uppsala, Sweden; 4 Department of Energy and Environment, Chalmers University of Technology, 412 96 Göteborg, Sweden; 5 Department of Animal Breeding and Genetics, Swedish University of Agricultural Sciences, Box 7023, 750 07 Uppsala, Sweden; 6 Defence Analysis, Swedish Defence Research Agency (FOI), 164 90 Stockholm, Sweden; 7 Secretariat of the Cross-Party Committee on Environmental Objectives, Karlavägen 100A, 103 33 Stockholm, Sweden; 8 Department of Social Anthropology, Stockholm University, 106 91 Stockholm, Sweden; 9 Department of Clinical Sciences, Swedish University of Agricultural Sciences, Box 7054, 750 07 Uppsala, Sweden

**Keywords:** Food security, Global challenges, Research priorities, Studies of future

## Abstract

To increase the awareness of society to the challenges of global food security, we developed five contrasting global and European scenarios for 2050 and used these to identify important issues for future agricultural research. Using a scenario development method known as morphological analysis, scenarios were constructed that took economic, political, technical, and environmental factors into account. With the scenarios as a starting point future challenges were discussed and research issues and questions were identified in an interactive process with stakeholders and researchers. Based on the outcome of this process, six socioeconomic and biophysical overarching challenges for future agricultural were formulated and related research issues identified. The outcome was compared with research priorities generated in five other research programs. In comparison, our research questions focus more on societal values and the role of consumers in influencing agricultural production, as well as on policy formulation and resolving conflicting goals, areas that are presently under-represented in agricultural research. The partly new and more interdisciplinary research priorities identified in Future Agriculture compared to other programs analyzed are likely a result of the methodological approach used, combining scenarios and interaction between stakeholders and researchers.

**Electronic supplementary material:**

The online version of this article (doi:10.1007/s13280-013-0417-3) contains supplementary material, which is available to authorized users.

## Introduction

Many global challenges face the present and coming generations, including food security, health, uneven distribution of wealth and resources, climate change, resource scarcity, and environmental degradation (e.g., MA 2005; IPCC 2007; Rockström et al. 2009; Godfray et al. 2010). The challenges vary between geographical areas and nations. Many of them relate to agriculture. This raises questions about how to sustain and improve production of crops and livestock to a growing population in a changing climate, the consequences of which we cannot fully anticipate, while maintaining resources and preserving the global environment for future generations (e.g., Vitousek et al. 2009; Beddington et al. 2012).

The future for agriculture and food security is an integrated part of the overall sustainable development agenda (UN 2000; Beddington et al. 2012), although the direction of change does not always seem to support a development that is sustainable. The achievement of sustainable development requires that its economic, environmental, and social components are integrated at all levels (WCED 1987). Regarding food security per se there is a widely accepted definition by FAO (1996), including dimensions like food availability and utilization. Additional aspects like food sovereignty and households’ food acquisition are also discussed in this context (e.g., Pinstrup-Andersen 2009).

The importance of taking action to support sustainable development is often stated in discussions and debates, but opinions on what, where, when, and how much to change differ between stakeholders. The arguments may also differ depending on the stakeholder’s perception of the future. Humans have always had an interest in speculating, divining, and opining about the future (Flower 2007), but it is obviously impossible to undertake empirical studies (in the strict sense) of the future. Several methods have, however, been devised to discuss the future in a structured, systematic, and scientifically organized way and to create and share pictures of the future (e.g., Dreborg 2004; Alm et al. 2012). In this article, such work is called “studies of future.”

With a set of possible future scenarios as the starting point, our preparedness to meet tomorrow’s challenges is likely to increase. In turn, this can help researchers to formulate cutting-edge research hypotheses, and guide policy makers and funding bodies to support future-oriented research. As issues related to food production and land use are complex, global and regional scenarios created to stimulate discussion and thinking about future agriculture need to be multifaceted. The overall aim of this study was to develop a research program on future agriculture contributing to food security 2050 by a systematic and coherent approach. The specific objectives of this article are (i) to describe and discuss the methodology that was applied, (ii) to present the results of the process, i.e., the scenarios, challenges, and research issues, and (iii) to compare the identified research priorities with some existing research programs within, or including, similar topic areas. Our hypothesis is that research programs developed using future scenarios as entry point will identify more cross-cutting and multifaceted research issues calling for multi- and interdisciplinary research.

## Studies of Future

Various methods exist to study the future, such as (i) historically based future studies, (ii) extrapolation of existing trends, and (iii) development of scenarios by various methods. Myrdal’s (2008) study on global development and rural communities in the Nordic countries in a 50 years perspective is an example of historically based future studies. Projections drawing on extrapolating current trends are often used for short-term perspectives, e.g., related to economics and markets. However, too many uncertainties intervene when projecting several decades ahead, and for those situations other approaches for scenario construction and analysis are commonly used. Scenarios were, for example, developed in the Millennium Ecosystem Assessment (MA 2005) to examine the delivery of ecosystem services to society in different futures, by the Intergovernmental Panel on Climate Change (IPCC 2000) for different climate scenarios and socioeconomic settings, and recently by the UK Ecosystem Assessment for providing knowledge of how to estimate the value of ecosystem services and the natural environment to the UK Society (UK NEA 2011).

A method employing four scenarios conceived from drivers along two axes, known as a scenario cross, was used by IPCC (2000). The scenarios were developed as four narrative storylines where the main factors which propel the scenario development were clustered in two dimensions, e.g., global versus regional, and strong environmental policy and rapid technological development versus weak environmental policy and slow technological development. The method using four scenarios organized along two axes has also been used in, e.g., the Millennium Ecosystem Assessment (MA 2005) and within research programs on forest futures (Sustainable Forest Management Network 2010; Future Forest 2012). Often, as in MA (2005), the final choice of two axes is based on an in-depth analysis of important drivers for the processes under consideration. The UK Foresight study on the Future of Food and Farming (Foresight 2011) used modeling of the food system to create scenarios along the two axes of economic growth (“optimistic” and “pessimistic”) and climate change (based on IPCC scenarios), but included more than two scenarios along the climate change axis.

In the Swedish Environmental Protection Agency’s future study called “Sweden year 2021” desired conditions in the future were envisaged and then the steps needed to achieve these conditions were defined (SEPA 1998). This method is known as back-casting (SEPA 1998). Visions of what is desired were also the basis for the scenario methodology applied within a Swedish research program on sustainable food production called “Food 21” (Sonesson et al. 2003; Gunnarsson et al. 2009). Target scenarios were formulated and alternative production and management systems were designed for pork, beef, milk, and potato production to meet different goals, e.g., product quality, animal and human welfare, resource use efficiency, and environmental protection (Gunnarsson et al. 2005; Kumm et al. 2005; Stern et al. 2005; Wivstad et al. 2005). The possible effects of different scenarios were evaluated using life cycle assessment (LCA) and economic calculations.

Morphological analysis is a method used for developing future scenarios (Zwicky 1969; Ritchey 2011), which makes it possible to analyze complex and multi-dimensional problems including both quantitative and qualitative factors (Carlsen and Dreborg 2008; Ritchey 2011). Morphological analysis permits very complex problem areas to be disassembled into different components which can be analyzed piece by piece and then combined into different scenarios (Stenström 2013). With morphological analysis all possible and conceivable alternatives are considered systematically. The number of scenarios is optional. This method was used by the Agrimonde project to provide the starting point for quantitative modeling of its two scenarios (Paillard et al. 2011). Morphological analysis was also chosen for developing the six scenarios in the UK National Ecosystem Assessment (Haines-Young et al. 2011; UK NEA 2011), because it made it possible to create a set of scenarios with a greater degree of differentiation than the traditional 2 × 2 scenario cross (Haines-Young et al. 2011).

Different methods for studies of the future have different pros and cons depending on the purpose. If the aim is to prepare for a wide range of conceivable futures the back-casting methodology might be less useful as it does not prepare for non-desirable developments. Which method that is best to choose for studying the future depends not only on the aim of the study (e.g., to guide actual planning or to stimulate research) but also on the topic’s complexity and time horizon (Dreborg 2004). For example, the search for the most likely future is less relevant when there is a high degree of structural uncertainty.

## Creating Scenarios for Future Agriculture

### Global and Regional Scenarios

In this study, morphological analysis (Zwicky 1969; Ritchey 2011) was used to develop scenarios, following the methodology described in detail by Stenström (2013). The rationale for selecting this method was that it allows for full traceability of all the considerations made during the process of creating scenarios, and for clear exposition of the assumptions in the scenarios (Haines-Young et al. 2011). Morphological analysis also makes it possible to analyze connections between the different quantifiable and qualitative factors without fully understanding the nature of causal relationships between factors; however, the states of the various factors have to be compatible within each scenario. All scenarios are comparable as they are constructed from the same factors, each explicitly described by a range of possible future states (modes of existence or manifestations). With morphological analysis it is possible to visualize and compare all the scenarios in a chosen set, to get a balanced whole.

Changes and adaptations within crop and livestock production and changes in land use are slow processes that may stretch over decades. Research on food production thus has to take a long-term perspective. Consequently a time horizon of 40 years (2050) was chosen. Our analysis started with identifying areas of key importance for future agriculture nourishing the world. To this aim, an expert group of researchers with the following expertise was recruited to the process: agronomy, soils science, ecology, veterinary medicine, animal science, agricultural economy, demography, peace and development, and energy and environment. The experts came from four different universities. The expert group had three 2-day sessions during a period of 6 months, working in an iterative process including facilitated and computer-aided morphological analysis (Ritchey 2006), back office editing of discussion notes in smaller groups and narrative writing to develop the scenarios. After each session a process report was distributed to the experts as well as homework (literature studies) to be done.

**Fig. 1:**
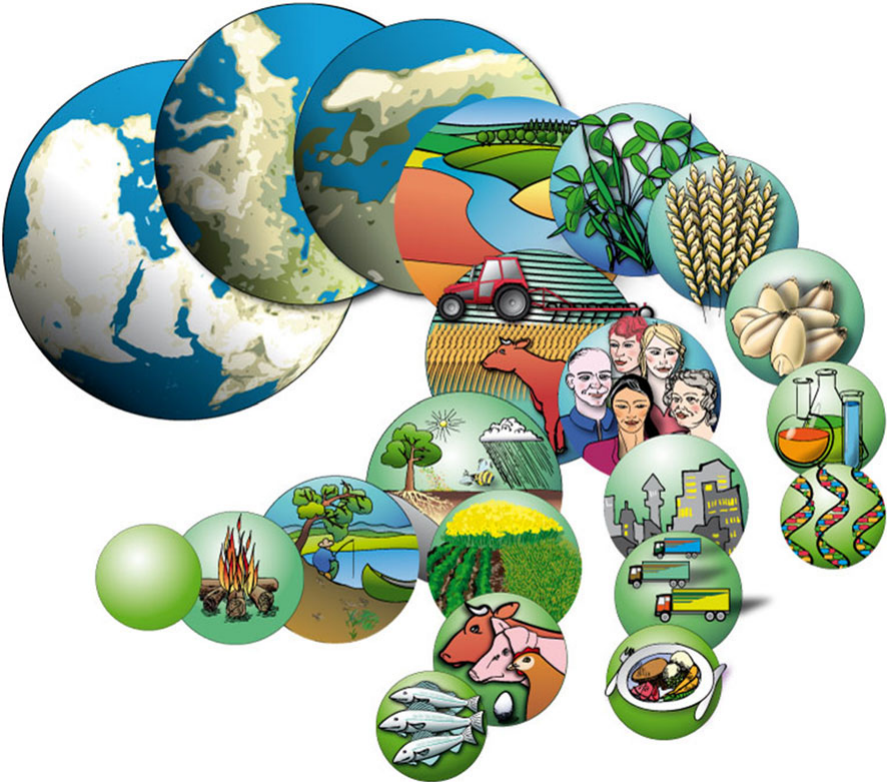
The scenarios were first drafted from a global perspective and then further developed on regional scale focusing on Europe based on a range of factors

Five scenarios were constructed as this number was regarded to be possible to handle and still generate the desired diversity. The scenarios were called “An overexploited world,” “A world in balance,” “Changed balance of power,” “The world awakens,” and “A fragmented world.” The scenarios were first drafted from a global perspective (Fig. 1). Each scenario was then further developed on regional scale focusing on Europe. Four of them were also later developed for sub-Saharan Africa (Magnusson et al. 2012). The five global and European scenarios developed for 2050 are described in detail in Öborn et al. (2011). Short versions of the scenarios are presented in Box S1 in Electronic Supplementary Material.

### Factors Forming the Scenarios and Their States

Here, the factors used in the scenarios are briefly described and the main sources for the elaboration of the states are given. A full description of all included factors is given in Öborn et al. (2011) and in the Electronic Supplementary Material (Appendices 1a, b).

**Fig. 2:**
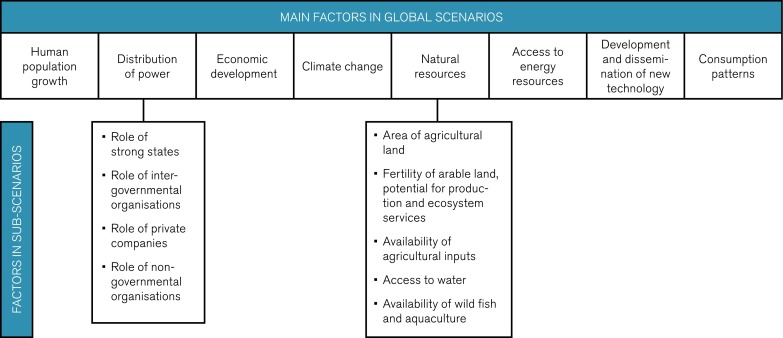
Factors analyzed in the global scenarios

The process started with identification of relevant factors to describe the global and the European situation from the perspective of future agriculture. Thereafter, a range of future states were identified for each factor in two morphological models (matrices), one global and one regional. Various factors that could have major impact on global development including future agriculture were proposed and scrutinized by the expert group in an iterative process. Eight main factors that can assume different states were identified and used in the global scenarios (Fig. 2). For each factor, three to six such states were used in the scenarios. The different states for the factors “Distribution of power” and “Natural resources” were generated as sub-scenarios in separate models. Several aspects of vital importance for future agriculture were included within “Natural resources” such as land area used for agriculture, i.e., grazing areas and land for crop cultivation (arable land), access to water, production potential, ecosystem services, soil fertility, access to agricultural inputs, and availability of wild fish and aquaculture.

Each scenario included all eight global factors. The scenarios were designed to express a significant variety, i.e., to cover as many states as possible, while still maintaining the internal logics of each scenario. The scenarios were created in a trial-and-error process using and revising the morphological models. The first step was to create scenario skeletons that formed an interesting and challenging whole. This was done by the expert group in a creative and structured way. The internal logic for each single scenario was checked, as well as the plausibility, and the scenarios were then compared with each other and revised in an iterative process. The second step was to write narratives from the chosen scenario skeletons. This was done by one member of the expert group, using relevant references supplied by the other members. In this step, internal inconsistencies and logical flaws in the individual scenarios were discovered. The third step was to revise the scenario skeletons jointly and to revise the narratives individually. The scenarios were also scrutinized by experts external to the group by the end of the development process.

**Fig. 3:**
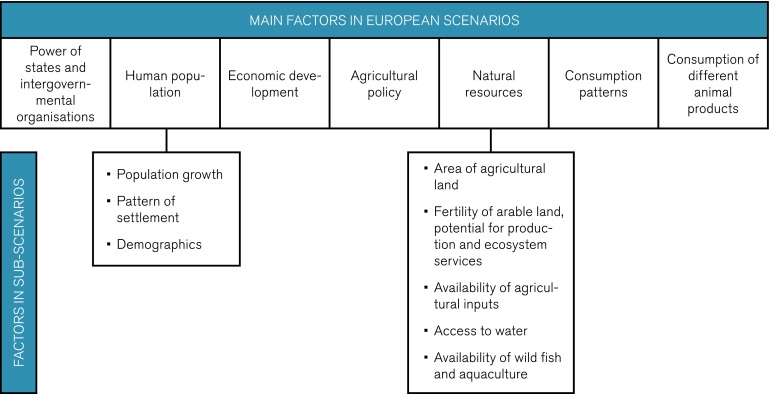
Factors analyzed in the European scenarios

Based on the global scenarios, corresponding regional European scenarios were constructed, including additional regional factors related to urban and rural development, agricultural policy, and consumption of animal products. Each regional factor assumed two to five states (Fig. 3). In the European scenarios, the factors “Climate change,” “Access to energy resources,” and “Development and dissemination of new technology” were given the same states as in the global scenarios, because these factors were considered to depend mainly on global development. Regional sub-scenarios were developed for the factors “Human population” and “Natural resources” in separate morphological models.

The different states for the factor “Human population growth” by 2050 in the global scenarios (8000, 9000, or 11 000 million) were derived from UN information and forecasts (UN Population Division 2011a, b, c). For human population growth in Europe, migration was considered an important component (Salt 2006). Climate refugees or immigrants in search of work thus constitute a main cause of the large population increase in Europe in some scenarios. The European scenarios also give an account of where people live, how urban and rural areas are developed, and how developed the infrastructure in rural areas is (Reginster and Rounsevell 2006).

Future global and regional “Distribution of power” has been much discussed in the literature, but the time perspective is often shorter than that used in this study (e.g., Dadush and Stancil 2010; Fogel 2010). Notably, not only the balance between states but also intergovernmental bodies (regional or international), large companies, and NGOs, as well as religious and ethnic movements were considered. Global power relations, i.e., a unipolar world order with one dominating center, a multipolar world order where all regions including Africa are developing, and fragmentation leading to regional and/or national protectionism, were taken as the starting point and a number of different combinations of power relations were taken into account. The combinations considered to be most likely for each scenario were used.

It is difficult to produce credible long-term scenarios for “Economic development” and we chose to use high versus low economic development and to differentiate between the global south and north (Appendix 1a in Electronic Supplementary Material). Bagnoli et al. (2005) at OECD published a forecast leading up to 2030 where three scenarios were simulated. They had high, moderate, and low economic growth based on, among other things, population forecasts from the UN. In IPCC (2000), gross domestic product (GDP) scenarios for the next 100 years for four scenarios were presented based on different storylines, and the UK Foresight (2011) used two economic scenarios, one with high and one with low economic growth.

The factor “Agricultural policy” was included in the European scenarios since the European Common Agricultural Policy (CAP) has large impact on global trade with agricultural products through the support of European farming and farmers. It is actively discussed both internationally (OECD 2010) and regionally (European Parliament 2010) and this naturally affects the development of agriculture, as well as food security, climate, and environment. However, the time perspective in these discussions is often considerably shorter than the 2050 perspective.

Existing global and regional climate scenarios for 1990–2090 were called on (IPCC 2000, 2007) to set the state of the “Climate change” factor. Three states have been elaborated on in this study; minimum (less than 2 °C increase), medium (2–3 °C increase), and maximum (3–4 °C) effects on the global temperature, followed by sea level rise, changed precipitation patterns and altered hardiness zones. The climate scenarios in the report from the Swedish government’s commission on climate and vulnerability (SOU 2007) provided a basis for the European scenarios.

Access to “Natural resources” such as agricultural land for grazing and crop cultivation, fresh water, fish, and different ecosystem services is projected to be of greater importance in the future than at present, but the demand and pressure can be higher than the sustainable capacity. This is included in the states of the Natural resource sub-factors that are integrated into sub-scenarios ranging from “less availability of all natural resources, except land area” and “weak biological systems and use of plentiful agricultural inputs” to “good availability to all resources.” These issues of availability and access to natural resources including ecosystem services have been discussed in the Millennium Ecosystem Assessment (MA 2005) and in reports from, for example, the OECD-FAO (2009). The global land resources required for agriculture is discussed and presented in the World Development Indicator (WDI 2009). Likewise, required land resources have been discussed and presented in EU (EC 2010) and Sweden (SCB 2010). The access to inputs in agriculture is thoroughly analyzed in the literature, for instance the finite resource of available phosphorus (Cordell et al. 2009; Vaccari 2009). The access to various ecosystem services in the future is elaborated in the Millennium Ecosystem Assessment (MA 2005) and by Rockström et al. (2009).

The factor “Energy resources” in the future has been thoroughly examined in several publications (e.g., WEC 2007; Aleklett et al. 2010; OECD 2010). Energy resources will not run out in the period up until 2050, but energy is likely to become more expensive and the balance between different types of energy sources may change (Brandt et al. 2010). Moreover, energy availability and costs may also be affected by power relations and regional conflicts (Correle and van der Linde 2006). Two important factors that may have a large impact on future energy prices are climate policies, that may increase the cost of fossil fuels substantially, and technological development. Technological development may both reduce the cost of extracting scarce resources of fossil fuels, but also provide cost-effective alternatives to fossil fuels such as wind power, biofuels, or nuclear energy. The states of global energy supply chosen in this study are related to land area required (large or small), the availability of energy sources (readily or scarce) and the price of energy (high or low).

The factor “Technological development” in sectors relevant to agriculture, such as biotechnology and resource cycling technology, is difficult to project (IAASTD 2009). When constructing the scenarios it was assumed that such developments could happen almost instantaneously or little by little, and independently of past occurrences be evenly or unevenly distributed at a global level. This factor is not included in the European scenarios as it is regarded to be an overruling global development. We have chosen to use the states rapid versus slow technological development in combination with how well the technology is distributed (even versus uneven).

Global and regional patterns of “Consumption of food” have been published by the FAO (2009) since the mid-1960s. There are also forecasts for 2030 based on FAOSTAT (WHO 2003). These reports have served as the basis for setting the state of this factor including the relation between plant- and animal-based products in the diet. In the European scenarios, there is a specified factor for “Consumption of animal products” as animal production can have a large impact on the environment.

## Identifying Future Challenges and Research Questions

### Scenarios as Starting Point for Identifying Knowledge Gaps

**Fig. 4:**
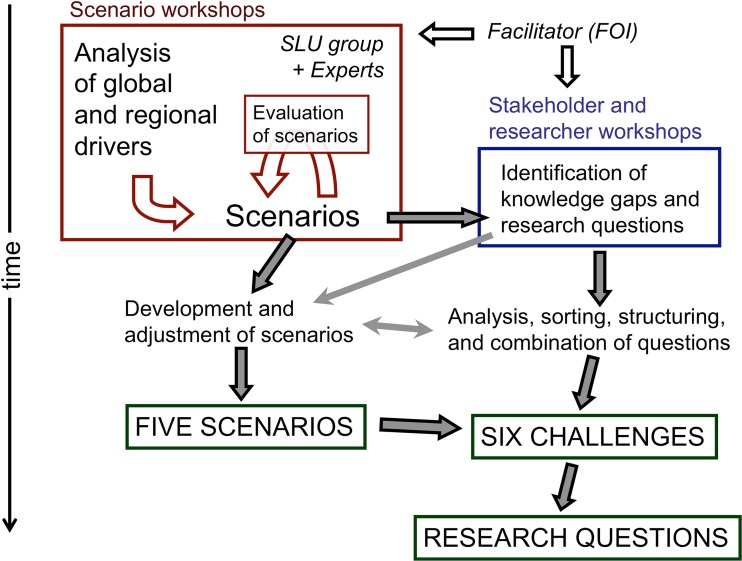
Illustration of the process through which six challenges for future agriculture were identified and research issues and research questions related to these challenges were formulated

The scenarios were taken as starting points to identify and discuss future demands and gaps in knowledge, and emerging research issues. Three stakeholder workshops, each comprising of 25–30 invited participants, followed the scenario work (Fig. 4). In the first workshop, we brought together representatives from the agricultural sector, governmental authorities, and non-governmental organizations from different areas of interest and responsibility related to agriculture, rural affairs, and the environment. In the second workshop, we gathered researchers from different disciplines and career stages, while the third one was devoted to young researchers only. The workshop participants had not been involved in the outline of the scenarios. The reason for inviting different categories of participants to the three workshops was to get a broader spectrum of experiences, expectations, and views on challenges and knowledge gaps for future agriculture (not to analyze them separately or to compare them).

As preparation for the workshop, short scenario descriptions were sent out to the invited participants some days prior to the workshop. Starting out a brief introduction was given where after the participants were divided into subgroups of 5–6 persons (mixed by affiliations) for two rounds of discussions with documentation on flip charts and reporting back in the plenary session after each round. In addition, one person per group was assigned to take notes during the discussion and collect all statements, questions, and reflections as well as flip charts. The subgroups discussed the following questions using two contrasting scenarios as starting point: (1) What challenges and knowledge requirements do you perceive for future agriculture, after having read the different scenarios? (2) What are the greatest opportunities and threats described in the scenarios with regard to future agriculture? (3) What knowledge requirements will these generate? (4) Within which areas is new research needed? (5) Within which areas can we exploit previous knowledge and experience? Different groups worked with different pairs of scenarios, but each scenario was handled by several groups.

The results of the three workshops (plenary reports, collected flip charts, and the notes from the group “observer”) were brought together, analyzed, and synthesized at two separate working meetings in which members of the expert group who had drawn up the scenarios participated. Based on the outcome of this process six critical challenges (research areas) for future agricultural research were formulated. Some of these were highly relevant for all scenarios; others were more relevant only for some of the scenarios. Within each challenge several research questions were identified. The different steps in the process are summarized in Fig. 4.

### Challenges and Research Issues

With the five scenarios developed for 2050 as a point of departure for the discussions, six challenges were identified:

- Reduction of the environmental impact of agriculture and mitigation of climate change
- Adaptation of agriculture to a changing climate
- Management of present and potential risks
- Responses to societal values and contribution to policies
- Agriculture and rural development
- Resolution of conflicting goals of agriculture and land use

Within each of the six challenges some broad major research questions were identified (listed in Box 1). The research needs are described below and are further elaborated in Bengtsson et al. (2010).

To reduce agriculture’s impact on the environment and to mitigate climate change, research is needed on efficient production systems that combine high production with low use of resources. In particular, how can recycling of nutrients, water, and wastes become more efficient? What is the potential of new innovative technologies, and how can various ecosystem services be used more efficiently? It is also important to find out by which methods agriculture can mitigate climate change, land degradation, and other effects on the environment. The consequence of the continuing structural transformations in food production systems on the environment and climate is another important issue. Some of the most important questions relate to the effects of different consumption patterns on food production systems and their effects on the environment and climate change.

The adaptation of agriculture to changing and more variable climatic conditions in combination with an increasing global population and food demand calls for new knowledge regarding the vulnerability, adaptability, and resilience of different production systems. The resource use efficiency and productivity need to be enhanced, while at the same time improving and maintaining ecosystem services, biodiversity, animal welfare, etc. Climate change adaptation of agriculture requires developing knowledge within many fields, for example, more efficient water use and recycling solutions, new crops and cropping systems, genetically improved animal breeds, improved protection against new diseases and pests, as well as how farmers adapt to variable and changing conditions.

To manage actual and potential risks knowledge about robustness and resilience of different production systems to different types of change and extreme situations is needed. For instance, readiness for extreme weather events caused by climate change and other ecosystem changes or collapses is crucial for maintaining food production. The danger of emerging trans-boundary animal and plant diseases as well as of zoonotic pandemics is also important areas to consider for research in the context of food security and safety. Research on the role of poor food security as a catalyst for social unrest and local conflicts is needed to increase our understanding and contribute to policy and action for alternative options. From a policy perspective, the use of or refusal to use new agriculture technologies (e.g., biotechnology) and farming systems is critical for the future, and a field where scientific analyses may contribute substantially.

To better understand values in society with respect to food production and accessibility, and to contribute with knowledge for policy, studies need to look into ethical issues related to food accessibility for households, food production, and its effect on the environment, for example, imports of cheap food and feed and export of pollution. It is important to consider the normative status (right or wrong) of different production methods and systems and also to compare different sets of values related to agriculture and food and the consequences of these values. Studies of processes leading to international agreements and political decisions (or lack of decisions) concerning agriculture and food production are central. The effects of different policy instruments, for example on land use, are also important to study.

The challenges related to agriculture and rural development require deeper knowledge of the interrelations between rural development and agricultural land use. This calls concurrently for deeper knowledge of the socio-economic organization of agricultural production and land use, and for knowledge of the drivers and barriers for living and working in very different rural areas. Questions of land ownership, labor demand, natural resources governance, and synergy effects of production with other aspects of the rural economy are central, as well as questions of quality of life in rural areas, not in the least in relation to urban areas.

To resolve conflicting goals related to agriculture and land use, research is needed on how people deal with situations in which different goals trade-off against each other, or in which people due to their different value systems and perspectives reach different conclusions. This may apply to issues of production intensity, ecosystem services and biodiversity, technologies, climate and environmental effects, animal and human health, as well as land use and land ownership relations. At the center of such analyses lies an understanding of how different human values and ideologies influence the means and methods for managing and resolving conflicts. Some apparent conflicts may be possible to transform into synergies. For example, better understanding of agroecosystem multifunctionality might lead to better management of ecosystem services combined with ecological intensification (Bommarco et al. 2013) to increase the productivity, climate mitigation, better water quality, and rural development. Of particular importance for the future global food production is how conflicts over water resources regionally and locally can be resolved. Another important area of conflict that needs more research to resolve is that between agriculture and urbanization—more than half the human population now lives in cities and the proportion is expected to increase. How can this be reconciled with the need for increased food production?

## Comparison with Other Research Programs

The list of research areas and questions identified through the process described above was compared with five other research programs partly or largely relevant for food security and agricultural production. The selected programs were: Strategy and Results Framework for the Consultative Group for International Agricultural Research (CGIAR 2011), Agrimonde (Paillard et al. 2011), the UK Foresight (Pretty et al. 2010), the European Union Framework Program 7 (FP7) (EC 2006), and the Swedish Governmental Research Bill (2008). The programs were selected to represent global, regional, or national perspectives (Table 1). In addition, we wanted to compare programs having different ownership, being endorsed by democratic institutions or by other organizations and having different types of origin, i.e., by and for whom they were developed.

**Table 1:** Selection criteria for the research programs included in the comparison

Program	Agriculture focus	Perspective	Ownership	Origin
CGIAR	Strong	Global	International organization	International organization
UK Foresight (Global Food and Farming Futures)	Strong	Global	Governmental agency	Academia^a^
Agrimonde	Strong	Global	Governmental agency	Academia
EC-FP7	Part	Regional	European parliament	European commission
Swedish Research Bill	Weak	National	National parliament	Government

^a^Part of the UK government Foresight program and reported to the UK Government Office of Science, but mainly comprising scientists

### Brief Description of Selected Research Programs

The six research programs represent different types of undertakings (Table S1 in Electronic Supplementary Material). The Swedish Research Bill (2008) was written for national use and EC-FP7 (EC 2006) for the European Union. The other programs have a global perspective, although CGIAR (2011) focuses on developing countries. In addition, the Swedish Research Bill and EC-FP7 cover research in general, whereas the other programs are specialized on agricultural research.

The Swedish Research Bill and EC-FP7 are political documents endorsed by parliaments and CGIAR is approved by the CGIAR Fund Council, a body of donors and stakeholders such as the World Bank and governmental and private sector development institutions, mainly in high income countries. Furthermore, CGIAR and EC-FP7 were written to fulfill goals that have been set outside the programs, i.e., the Millennium Development Goals (MDGs) and Lisbon EC Council meeting’s goals, respectively. In contrast, UK Foresight (Pretty et al. 2010), Agrimonde (Paillard et al. 2011), and Future Agriculture were initiated by researchers and here the stakeholders are farmers, agro-industry, consumers, citizens, etc. The Swedish Research Bill and EC-FP7 are directly governing research funding. They cover a specific, short time period (4 and 7 years) but no time table is presented for the outcome and future challenges are described without putting them into a time perspective. The time perspective of Agrimonde, UK Foresight, and Future Agriculture is several decades (until 2050), whereas CGIAR has a shorter time perspective (until 2025).

The CGIAR program targets agriculture in low income countries with the overall aim to combat hunger and poverty. In the development framework where the CGIAR acts, the overall priority setting is from the MDGs and expressed as four “system level outcomes”: (1) improving food security, (2) reducing rural poverty, (3) reducing undernourishment, and (4) sustainable management of natural resources. From these priorities, the research community within the CGIAR and their associates have identified research issues and generated thematic research programs based on the global challenges and the areas of expertise of various CGIAR centers. Finally, these thematic research programs have to be approved by the CGIAR Fund Council.

Pretty et al. (2010) summarize the research questions emerging from the UK Government’s Foresight Global Food and Farming Futures project (Foresight 2011), which was initiated to meet the challenge how to feed an expected population of some nine billion by the mid-twenty-first century. A major aim was to direct research to issues that influence current and future policy frameworks and which are relevant to the needs and issues of farmers and agriculturalists in different parts of the world. The Foresight study on the Future of Food and Farming (Foresight 2011) used quantitative modeling of the food system to create scenarios along the two axes of economic growth and climate change. The scenarios were not explicitly used to envisage different futures, but rather formed the basis (“framed the discussion”) for identifying five major challenges for the future world food system and policy recommendations. While the Foresight document has a long-time horizon (to 2050), many of the questions in Pretty et al. (2010) have a shorter time frame. It is furthermore not clear how these questions relate to the scenarios. Rather, the horizon-scanning approach used by Pretty et al. (2010) seems to have been a structured “expert anticipation” process largely based on present trends, indirectly anchored in the quantitative scenarios of the Foresight (2011). The process involved a large number of leading experts and representatives of major agricultural organizations around the world, but mainly from UK and English-speaking nations.

Agrimonde (Paillard et al. 2011) was mainly developed by a French group based at CIRAD-INRA, involving a panel and a project team to a large extent representing research and high level stakeholders. Starting with a morphological analysis to reduce the complexity of the food and agricultural system, it subsequently modeled possible states of the world’s agricultural production based on two contrasting scenarios: (1) the Agrimonde GO (the Millennium Ecosystem Assessment Global Orchestration), which is a trend-based scenario where liberalization and technological progress play major roles; (2) Agrimonde 1, which is based on sustainable development, environment-friendly agriculture and reduction of inequalities. The latter is a normative scenario exploring sustainability as an overarching goal. The research questions were derived from a narrative discussion of the two scenarios. The relations between the research questions and the two views of the future are in general logical but not always clear from the text, and the relative importance of different issues is not clearly stated.

The Seventh Framework Program (FP7) of the European Community (EC) for research, technological development, and demonstration activities (2007–2013) was approved by the European Parliament and Council (EC 2006). The objective is to strengthen the scientific and technological bases of the EC industry and thereby ensuring a high level of competitiveness at an international level. The overall aim is to achieve the strategic goal set by the EC: “to become the most competitive and dynamic knowledge-based economy (i.e., education, research, and innovation) in the world, capable of sustainable economic growth with more and better jobs and greater social cohesion.” EC-FP7 has an overarching aim to contribute to sustainable development. For cross national research 10 themes were determined, including areas such as health; food, agriculture and fisheries; biotechnology; energy; environment including climate change; and socio-economic sciences and humanities. Based on EC-FP7 different priority areas for support are being further developed into Calls for proposals that are issued annually.

The Swedish governmental bill on research and innovation was proposed by the government and approved by the parliament (Swedish Governmental Research Bill 2008). The Ministries of Finance and Education were responsible for drawing up the bill with contributions from other ministries. Governmental bodies such as research agencies, universities, and authorities were requested to submit their research strategies, whereas other organizations, e.g., research foundations, academic organizations, industry, and non-governmental organizations, were invited to make suggestions about important research areas. Several official investigations, reports, and memoranda were also considered when drafting the bill and they had previously been sent out for consultation to many different stakeholders. The Swedish Research Bill is focused on disciplinary research and the main message is summarized with three key words: medicine, technology, and climate. The bill identified six strategic research areas: medicine and life sciences; illness of large importance for public health; technological research; research related to climate; security and preparedness; strategic research within social science and humanities. Three criteria were used when these areas were identified: research that can contribute to finding solutions to important global problems and issues, areas in which Sweden already carries out world-class research, and areas where companies in Sweden are carrying out their own research and development and where state investments could reinforce the development and competitiveness of the business sector in Sweden. Agricultural issues are found mainly within “Climate,” which includes energy, impact on natural resources, marine environment research, sustainable use of natural resources, and climate models.

### Similarities and Differences in Research Priorities

The studied research programs have different foci, which are expected as they differ in aim, ownership, and time perspectives (Table 1, Supplementary Table S1). When compared with respect to the six challenges identified in Future Agriculture, many similarities can be seen between the programs, but also certain differences (Table 2). In various ways they all suggest research on how to reduce environmental impact, and climate change mitigation and adaptation. In the EC-FP7, however, there is a lack of integration between agricultural production and environmental aspects, the former being included in the knowledge-based bio-economy (KBBE Theme 2) and the latter in a separate section about environment and climate change (Theme 6). In most programs, the area “management of present and potential risks” is dominated by dangers of zoonoses as well as other infectious diseases and extreme weather events.

**Table 2:** Comparison between Future Agriculture (FA) and the reviewed research programs, with regard to highlighted research issues related to agriculture, food production, and rural development

	CGIAR (2011)	UK Foresight (Pretty et al. 2010)	Agrimonde (Paillard et al. 2011)	EC-FP7 (2006)	Swedish Research Bill (2008)
Missing or less emphasized as compared to FA	Consumers’ role and values as well as policy making are not so prominent. Resolving conflicting goals between different land uses is not explicitly mentioned (but trade-offs between increasing productivity and maintaining and enhancing the provision of ecosystem services is included)	Missing: Resolving conflicting goals	Resolving conflicting goals emerges in several places, but it is not highlighted as a separate important issue (although governance is discussed)	Lacking integration of production and environment, i.e., separation of production research (bio-based economy) and issues related to environment and climate. Less emphasis on socio-economy and cross-disciplinary research. Resolving goal conflicts between different land uses etc.	Research on rural development in general is scarce (although entrepreneurship is included) and rural development related to agriculture is missing. There is no emphasis on resolving conflicting goals. Almost nothing about societal values and normative status of production systems. Agriculture’s response to societal values is missing as well as policies and laws for food production
Included in this program, but missing or less emphasized in FA	Research for provision of nutritious food and the importance of adding gender aspects into the research	Indicators of sustainability and the concept of sustainable intensification. Markets and prices. Ecosystem services, biodiversity, and resilience get more attention than in FA’s program	Ecological intensification. International trade in agricultural and food production. Relations health–food production	More focus on research on biotechnology and “omics” as well as production of bio-resources other than food, and on reducing imported energy through enhancing efficiency and renewable energy sources	Bioinformatics and new possibilities given by more powerful and larger computers and data banks. Bioterrorism harming agriculture and food production. Lifestyle-related diseases, old people’s way of living and rehabilitation issues

**Box 1:** Within each of the six challenges for future agriculture some broad major research questions were identified

**1. Reduction of the environmental impact of agriculture and mitigation of climate change**
How and by which methods can agriculture mitigate climate change?
How can agriculture mitigate land degradation and other forms of environmental pollution?
How can recycling of nutrients, water and wastes become more efficient?
What are the environmental and climate impacts of structural changes in agriculture—specialization versus integration, small scale versus large scale, and geographic localization?
What is the potential for increased efficiency and productivity by innovative technologies in agricultural production systems?
What are the environmental and climate impacts of different consumer preferences and consumption patterns?
**2. Adaptation of agriculture to a changing climate**
What are the vulnerability, adaptability, and resilience of different agricultural production systems?
Which functions in terms of ecosystem services do different species and biodiversity have in present and future production systems?
How can crop and livestock species and varieties/breeds be adapted to new climatic conditions (higher temperature, longer periods of drought, extreme weather events) and what is the potential for domestication of “new species”, e.g., to utilize marginal areas and organic waste?
How can resource use efficiency and production be increased on agricultural land while at the same time maintaining ecosystem services, biodiversity, and animal welfare?
Which management options and technologies exist to combat emerging pests and diseases in crop and livestock production?
How can integrated systems—at different scales—for crop, livestock, and energy production be designed and evaluated?
Which options for new land uses exist and what are the potential advantages and disadvantages of more land into different types of agricultural production?
**3. Management of present and potential risks**
What threats against food security do diseases and pests emerging in crops and livestock constitute, and how can they be managed?
How can threats against food security caused by climate change and other ecosystem changes or collapses be managed and avoided?
How does the use, or refusal to do so, of new technologies and farming systems affect food security?
What consequences does poor food security have for social unrest and local conflicts?
How do agricultural production systems constitute threats for ecosystem resilience, and affect risks of environmental collapse and climate-induced catastrophes?
How do agricultural production systems increase or decrease the risks of zoonotic pandemics?
**4. Responses to societal values and contribution to policies**
What is the normative status of different forms of agricultural production for food, feed, energy, etc., i.e., are they perceived as right or wrong?
Which different sets of values related to agriculture, food, and technology can be identified?
What are the consequences of different sets of values, with regard to the actions or the absence of actions of producers, consumers, and politicians?
How do political processes lead to international, regional, and national agreements, policy instruments and laws supporting or restricting agricultural land use and production, e.g., climate, environment, biodiversity, trade, rural development, animal health, and welfare?
What are the effects and consequences of various international agreements, policies, and laws on agricultural production and land use?
**5. Agriculture and rural development**
How do changes in agricultural and food production systems affect rural communities and rural economies?
What effect does increased competition for land-based resources have on producer prices and the economy in the agricultural sector, e.g., more large-scale and specialized production, or integration of production in new kinds of ownership and collaboration?
What is the importance of different forms of land tenure, ownership, and collective action for agriculture and rural development?
How do urban and rural areas interact through flows of natural resources, goods, energy, ideas, capital, people and means of transportation?
How can economic and social sustainable development in rural areas and food security in cities be combined?
What are the effects of different policies on rural livelihoods and entrepreneurship?
How can knowledge developed on communication and collaboration be applied in agricultural production and natural resource management?
**6. Resolution of conflicting goals of agriculture and land use**
What are the conflicts and trade-offs between different agricultural land uses: conflicts between goals, different techniques or land management systems?
How should conflicts over water resources and water use regionally and locally be addressed and resolved?
What are the possibilities for resolving conflicts between urbanization and agriculture, e.g., urban planning, urban farming, small-scale production in urban/peri-urban areas
How can trade-offs and synergies among ecosystem services, production, climate impact, biodiversity, animal and human welfare, and health be identified and managed?
What are the possibilities for multiple-use and multifunctional systems to resolve conflicts in agriculture and land use?
How do human values affect the means and methods for managing and resolving conflict?

Research questions related to responses to societal values, which was highlighted in Future Agriculture, were given less priority or were missing in some of the other programs, although the UK Foresight paid much attention to markets, consumption, and agricultural development in relation to social issues (Pretty et al. 2010). Research on policy formulation and implementation are examples of other areas given less priority in most of the reviewed research programs. How changes in agriculture and food production systems affect rural communities and rural economies is another research issue identified by Future Agriculture that is weak or missing in several of the other research programs. However, entrepreneurship was emphasized in the Swedish Research Bill and small and medium sized enterprises were prioritized in EC-FP7. Research on resolving conflicting goals related to agriculture and land use was highlighted only in Future Agriculture. However, CGIAR included trade-offs between intensification of production and increasing productivity and maintaining and enhancing the provision of ecosystem services.

Some research areas prioritized in other research programs were less pronounced in Future Agriculture (Table 2). Food quality, provision of nutritious food, and human health are more emphasized in CGIAR, Agrimonde, and the Swedish Research Bill than in Future Agriculture. This is probably not a result of the methodology used per se, but rather a consequence of the composition of the expert group that created the scenarios (the Future Agriculture team did not include expertise in human nutrition and medicine). Some of the other programs are more explicit when it comes to the technologies to be developed in agricultural research, specifically including biotechnology, “omics” methodology and bioinformatics. Research on renewable energy and other non-food products was not included in Future Agriculture having the focus on food production. Gender was another aspect not specifically addressed within Future Agriculture. Ecosystem services, biodiversity, and resilience were included in Future Agriculture but given more attention in some other programs (UK Foresight and Agrimonde). This was likely because these programs had a narrower scope and did not cover factors such as power relations, global economic development, regional migration patterns, and urban and rural development as Future Agriculture did, thus allowing more space for developing various aspects such as the role of ecosystem services and biodiversity for the resilience of agricultural production.

Several of the research programs included in this comparison argues that a global perspective is needed to identify research to ensure food security, as there is only one common globe on which most resources are finite. Future Agriculture shares the global perspective with UK Foresight, Agrimonde, and CGIAR and all these programs focus on food security and the needs of the global human population. On the other hand, national and European political programs naturally place more emphasis on the needs of industry and citizens in their regions. In fact, EC-FP7 emphasizes agricultural non-food production rather than food production, and this is also the case in the Swedish government’s research policy. The aim of the latter is specifically to strengthen Sweden’s position as a research nation, thereby strengthening its capacity to compete in a global world to increase economic growth and welfare in Sweden (Swedish Governmental Research Bill 2008).

## Scenarios to Identify Future Research Needs

The purpose of this study was to identify research issues addressing challenges and opportunities related to agriculture and food security. The construction of scenarios is commonly regarded to be a useful way to examine possible futures at different scales, and to connect different disciplines in a discussion of the common future. Several recent scenario analyses related to agriculture, food security, and the sustainable use of natural resources exist (e.g., IPCC 2000; Carpenter et al. 2005; MA 2005; Sustainable Forest Management Network 2010; Cilliers et al. 2011; Foresight 2011; Future Forest 2012; Paillard et al. 2011; UK NEA 2011). These examples used different methodologies, but have several things in common: The use of scenarios allowed a more open discussion of possible futures from different perspectives, not simply assuming that the future is an extrapolation of (some of) today’s trends. Scenario analysis also forced the authors to focus on the needs in the future rather than today’s short-term objectives using an explicit and common picture of the future.

As stressed before, changes in agriculture have long lead-times. Therefore, research for future agriculture should ideally have a long-term perspective. Even so, the work for global food security faces similar management challenges as the global climate change negotiations. Regional and national politicians often choose to prioritize more current and immediate issues, and they avoid placing their descriptions of the future on a time scale (Andersson and Westholm 2012). Consequently, the time horizon of future needs is not identified in the politically decided programs EC-FP7 and Swedish Research Bill. The programs written by scientists in cooperation with stakeholders (Future Agriculture, Agrimonde, and UK Foresight) cover a long and defined time period (until 2050).

At present, agricultural research is heavily dominated by natural science and technology. The Swedish Research Bill illustrates that even programs aimed to cover all public research of a nation tend to focus on natural science and technology. As argued by Weiner (2003), agricultural research often seeks to address complex multi-facetted challenges with disciplinary research and experimental design. The research issues and questions defined based on the scenario work and stakeholder dialogues in Future Agriculture call, for strong involvement of researchers from all science domains, including social sciences, humanities, economics, and ethics. They also seem to reveal the need for interdisciplinary research to a higher degree than other programs (Table 2, Supplementary Table S1). This is illustrated by the 37 research questions raised by Future Agriculture (Box 1) that are structured under challenges rather than under scientific disciplines. Agrimonde (Paillard et al. 2011) also emphasizes inter/multi-disciplinary and broad questions related to the scenarios rather than disciplines, whereas the UK Foresight formulates five challenges but mainly emphasize disciplinary questions, albeit also in other disciplines than natural sciences (Pretty et al. 2010; Foresight 2011).

Scenarios seem to open up for a broader perspective, where humans (including political and socio-economic aspects) have a more prominent role in the program. For example, goal conflicts and knowledge increasing the ability to resolve these is highlighted in Future Agriculture but hardly mentioned in the other programs. Some of the other programs tend to rely more on technological innovations as measures for development. This is illustrated by the title of the Swedish Research Bill: “A boost to research and innovation.” Future Agriculture highlights to a larger extent the implementation of research results, which is related to societal values, consumers’ attitudes, and policy formulation.

The inclusion of non-agricultural factors in the Future Agriculture scenarios and the integration of the global and regional perspective influenced the challenges being identified as well as the research issues that emerged. Cross-cutting issues were identified of which some are going far beyond traditional agricultural research. This type of research requires expertise in a wide range of disciplines, including the humanities and socio-economics, as well as multidisciplinary teams and interdisciplinary research methods. The stakeholder involvement in identifying challenges and knowledge gaps using the different scenarios as entry point was vital for the outcome of the process as well as the 40-year time horizon. Existing academic structures did not limit the creativity and if implemented the research program will certainly vitalize future agriculture research and education benefitting food security and sustainable development.

## Concluding Remarks

This study illustrates that using scenarios for identifying future research issues resulted in a strong emphasis on the need of interdisciplinary research. The methodology also reduced the bias from the individual participating researchers’ disciplines. Based on the scenarios, six social and biophysical overarching challenges (research areas) with related research questions were formulated: (i) reduction of the environmental impact of agriculture and mitigation of climate change, (ii) adaptation of agriculture to a changing climate, (iii) management of present and potential risks, (iv) responses to societal values and contributions to policies, (v) agriculture and rural development, and (vi) resolution of conflicting goals of agriculture and land use.

The comparison with other research programs that include agriculture or related sectors showed that the research questions identified varied depending on the ownership and time perspective of the program. However, all programs highlighted issues like reducing the environmental impact of agriculture and climate change adaptation. In addition, most programs emphasized risks of infectious diseases and extreme weather events. The research issues identified in the Future Agriculture program were often more interdisciplinary than those of the programs compared with. More focus was put on societal values and the role of consumers in influencing agricultural production, policy formulation, and implementation as well as on resolving conflicting goals.

Hence, scenarios provided us with a context for a common identification of problems and knowledge gaps *before* suggesting solutions (research issues), which helped to broaden the discussion beyond special interests among researchers and stakeholders. The scenarios both helped to think in a longer time perspective and to identify research needs that are not on the public agenda, or perceived as warranted, but still are possible to occur. The use of scenarios also made it possible to analyze the interconnectivity between factors or drivers on global and regional levels. Discussing several diverse scenarios, including the undesired, can broaden the research issues and demonstrate the necessity of thematic approaches. The partly new and more interdisciplinary research priorities identified in Future Agriculture compared to other programs analyzed are likely a result of the methodological approach used, combining scenarios and interaction between stakeholders and researchers. Although it is difficult to prove, we are convinced that it would not have been possible to reach the results we did in such a short time and with the relative modest funding, if we had not used scenarios as a central point in the analysis.

## Electronic Supplementary Material

Below is the link to the electronic supplementary material.

Box S1 (PDF 21 kb)

Table S1 (PDF 63 kb)

Appendix 1a (PDF 72 kb)

Appendix 1b (PDF 71 kb)
