# Supplementary material for: Scenario Development as a Basis for Formulating a Research Program on Future Agriculture: A Methodological Approach
Source: Ambio. 2013 Jul 9;42(7):823–39. doi: 10.1007/s13280-013-0417-3 (PMC3790135; doi:10.1007/s13280-013-0417-3)
Supplement: Supplementary file 1 — Box S1 (PDF 21 kb) [file 13280_2013_417_MOESM1_ESM.pdf]

Box S1. Five scenarios for 2050 were developed from a set of defined factors using morphological analysis. The scenarios were first drafted from a global perspective, and each scenario was then further developed focusing on Europe (detailed description in Öborn et al. 2011)

An overexploited world. The climate is changing dramatically, with average global temperature projected to increase by 4-5˚C (1990-2090). Population growth has exceeded the UN forecasts and the population is 11 billion. The USA dominates the political and economic scenes. The Western world is achieving strong economic development but poverty prevails in large parts of the world, where poor food security results. Energy consumption is high and there are no strong climate policies. The demand for land resources is high owing to the increased world population and use of biofuels driven by energy security concerns. The land areas being used for agricultural production and livestock grazing expands. Soil fertility, water resources and ecosystem services decline as the result of overexploitation. As a result, food security is negatively affected.The proportions of animal and plant-based food are the same as they are today, both globally and in Europe. Europe has a protected market and well developed intergovernmental institutions. In this region the urbanization is even higher than at present and mainly centered on major cities.

A world in balance. Population growth has been slower than the UN forecasts and we are now 8 billion. Climate change is modest due to reinforced and efficient political activities; the increase in average global temperature is projected to remain below 2˚C (1990-2090). Economic development is strong in many regions of the world, including Africa. Powerful intergovernmental actors are reaching global agreements, for example about environmental issues. Rapid technological development in many sectors, including energy and agricultural, together with a wide and even distribution of new techniques, is a prerequisite of this scenario. Thus, pressure on land resources is relatively low. Soil fertility, productivity and the availability of ecosystem services are increasing, both in Europe and globally. Urbanization is high, but rural areas are also flourishing as a result of the development of businesses that are not dependent on proximity to cities. The proportion of animal-based food in the human diet has become lower in Europe than today, and fish and seafood consumption constitutes a higher proportion of the total food consumption than at present. Overall, global food security is likely to be relatively high in this scenario.

Changed balance of power. The global balance of power has been displaced towards India and China, where economic development is very strong. The global economy is characterized by deregulation and free trade and the European economy is stagnant.Global population growth has dipped below UN forecasts (8 billion in 2050), mainly due to rapid economic development in Asia slowing population growth. In Europe, population growth has been very high owing to migration. In this region urbanization is high and both large cities and small towns are expanding. Political commitments regarding climate and environment are limited and global average temperature is projected to increase by 4-5˚C between 1990 and 2090. Fossil fuels

(mainly coal) are readily available, and the prices are relatively low. Rapid technological development has led to new methods of production in many countries. The total agricultural land area is about the same as at present, but is being displaced towards the poles and the equator in response to climate change. The consumption of animal products has increased globally. In this scenario, global food security is relatively high but there are regional variations with some areas where food security is low, mainly as an effect of climate change.

The world awakens. After many years of limited commitments regarding environment and climate, the global community finally agrees on efficient policies. Global average temperature is projected to increase by 2-3˚C between 1990 and 2090. The balance of power is distributed among several centres: Brazil, China, Europe, India, North America and Russia. Population reaches the UN forecast of 9 billion in 2050. Environmental and human rights organizations have a more influential role. Fossilfuels and other energy sources that do not require land resources dominate, but forceful climate policies limit emissions. Environmental ambitions are high, so the rainforests are protected from deforestation. The availability of external inputs for agriculture is low, and prices are high. Agricultural land use in Europe has been redirected towards the East and the North following severe drought in the Mediterranean region. Both urban and rural areas are being developed. After an initial decline in food security globally, strong policy measures are subsequently reversing this trend.

A fragmented world. Population growth is high and exceeds the UN forecasts (11 billion in 2050). Global average temperature is projected to increase by 4-5˚C between 1990 and 2090. Due to weak intergovernmental actors and an absence of dominant nations, power relations are unsettled and international negotiations often fail. Private companies dominate the market. Technological development is slow and the distribution of new technologies is uneven. Fossil fuels (especially coal) dominate the energy market. The high food demand increases the need for agricultural land. Water resources are scarce, and soil fertility and ecosystem services are decreasing. In Europe, population growth has been very high following uncontrolled migration. This has led to high urbanization, with insufficiently controlled urban growth, both within city bodies and in the form of urban sprawl (uncontrolled expansion of urban areas into the countryside). The consumption of animal products is decreasing as the result of poverty, both globally and in Europe. In this scenario, global food security is relatively low and regionally it is dependent on local, national and regional conditions.
