# Supplementary material for: Scenario Development as a Basis for Formulating a Research Program on Future Agriculture: A Methodological Approach
Source: Ambio. 2013 Jul 9;42(7):823–39. doi: 10.1007/s13280-013-0417-3 (PMC3790135; doi:10.1007/s13280-013-0417-3)
Supplement: Supplementary file 2 — Table S1 (PDF 63 kb) [file 13280_2013_417_MOESM2_ESM.pdf]

Table S1. Short description of the reviewed research programs

	**CGIAR 2011**	**Foresight 2011 (Foresight 2011; Pretty et al. 2010)**	**Agrimonde (Paillard et al. 2011)**	**EC FP7 2006**	**Swedish Research Bill 2008**	**Future Agriculture**
Purpose of program	Overview of strategic thinking priority setting and main research issues within the Consultative Group on International Agricultural Research.	Encourage development of national and international policies. Improve dialogue and understanding between agricultural research and policy. Direct research at issues that influence current and future policy frameworks.	Present a strategic reflection on interactions between agricultural production, food and sustainable development. Guide research orientation in agronomy and food. Initiate debates on the national scale. Favor participation of French experts in international debates.	Strengthen the scientific and technological bases of the EC industry. To make EC the world's most competitive and dynamic knowledge-based economy (i.e. education, research and innovation). Sustainable economic growth.	Decide on distribution of public money governing activities of universities and other public research institutes.	Identify research needs for development of sustainable and efficient food production and land use systems. Strengthen coordination and cooperation within and between universities, research institutions and stakeholders.
Content of the program	Agricultural research Developing countries	Agricultural research Global	Agricultural research Global	All kinds of research Regional (EU)	All kinds of research National (Sweden)	Agricultural research Global/Europe
Time perspective in the program	Endorsed 2011. Time period to 2025.	Written 2009-10. Time period to 2050.	Work carried out 2006-2009. Time period to 2050.	Decided 2006. Covers 2007-2013.	Decided 2008. Covers 2009-2012.	Written 2010. Time period to 2050.
Regularity of program	Foreseen to be about 3-6 years	Unclear, probably a one-time event	Unclear, probably a one-time event	Every 6-7 years	Every governmental period (4 year)	One-time event
Process to create the program	Overall priority setting derived from the Millennium Development Goals, The research community within the CGIAR and their associates identified system level outcomes, research issues and generated research programs based on the expertise at CGIAR centers.	Initially five challenges based on scenarios and modeling of the food system (Foresight 2011), forming the basis for horizon-scanning approach. Research questions emerging from a structured "expert anticipation" process (Pretty et al. 2010). 100 questions in 14 themes selected from a larger set suggested by representatives for scientific societies, agricultural organizations, and academic institutions (dominance of UK and US).	Two contrasting scenarios used to identify challenges and research areas: one trend-based with liberalization and technological progress as major factors, one based on sustainable development. Scenarios initially based on morphological analysis and use of FAO data in simulations with Agriobiom model. Made by a working collective mainly based at CIRAD-INRA (France).	Approved by the European Parliament and Council (EC 2006). Process to identify priority areas and formulate program not described. National representatives (Governments) and experts (Research Councils) could provide input and feed-back on drafts and prior to annual calls through which the program is implemented.	Organized by government ministries. Research agencies, universities and authorities submitted their research strategies. Research foundations, academic organizations, industry and non-governmental organizations could submit important research areas.	Research challenges formulated by expert group based on future scenarios created by morphological analysis (see section 2), stakeholder and researcher workshops.

Stakeholders' influence on the program	The UN Millennium Development Goals was the basis for the program, which then was approved by the donor community funding the CGIAR.	Foresight report used high level stakeholders. Research questions written by "leading experts and representatives of major agricultural organizations around the world". Not much input from other stakeholders.	Project team representing research and high level stakeholders. Not much input from other stakeholders.	Determined by existing formal institutions in public and private sectors (research institutions, authorities, industry, membership organizations etc.).	Political decisions by parliament based on material collected from different stakeholders.	Stakeholders participated in a workshop where challenges and research issues were discussed (see section 2).
Traceability of priorities	Describes the reasoning from the MDGs, via the "system level outcomes" to the actual research priorities.	Not entirely clear. Unclear connections between challenges (Foresight 2011) and research themes and questions (Pretty et al. 2010). Each theme has a description of the considerations that were made and why.	A narrative discussion of the two scenarios leading to research questions, generally logical but not always clear and traceable. No priorities among the questions.	Overall goal clearly stated (from Lisbon EC Council meeting 2000). Not clear how research priorities were identified and included or excluded during the preparation of the draft(s).	The following criteria for priorities are: solve important global problems, public investments to strengthen industry, Sweden's development and competitiveness.	Creation of the scenarios described in Öborn et al (2010). Research program, with motives for identified challenges and research issues reported in Bengtsson et al (2010).
Disciplinarity	Interdisciplinary research important part. Thematic research causes, directed towards the four system level outcomes derived from the MDGs.	Focus on disciplinary questions and very broad general ones, few clearly stated interdisciplinary issues identified. No clear statements of importance of interdisciplinary research.	Broad and general questions highlighting inter/multi-disciplinary issues, research questions related to the scenarios, rather than disciplines.	A mixture of broad and complex research issues and more focused and narrow research questions. Thematic areas broader than academic disciplines but in most cases not interdisciplinary, some related to sectors and others to technologies, academic areas or cross-cutting issues.	Focused on disciplinary research. The general importance of interdisciplinary research stated a few times, but no clear examples of inter-disciplinary research questions are given.	Both intra- and interdisciplinary research questions needed to solve the raised research questions; the need for interdisciplinary research is highlighted.

Most important research areas	• Improving food security<br>• Reducing rural poverty<br>• Reducing under-nutrition<br>• Sustainable management of natural resources	• Natural resource inputs<br>• Agronomic practices<br>• Agricultural development<br>• Markets and consumption	• Ecological intensification<br>• Criteria for efficiency of production systems<br>• Interactions between food, consumption, health, and sustainable development<br>• Role of technology and innovation in agriculture<br>• Regulations and governance for sustainability and change in food consumption<br>• Local and rural development in developing countries<br>• Role of international trade in food production	Ten themes, most relevant were:<br>• Health<br>• Food, agriculture and fisheries, biotechnology<br>• Energy<br>• Environment (including climate change)<br>• Socio-economic sciences and humanities<br>• Security	• Medicine and life sciences<br>• Illness of large importance for public health<br>• Technological research<br>• Research related to climate<br>• Security and preparedness<br>• Strategic research within social science and humanities	• Reduction of the environmental impact of agriculture, mitigation of climate change<br>• Adaptation of agriculture to a changing climate<br>• Management of present and potential risks<br>• Responses to societal values, contribution to policies<br>• Agriculture and rural development<br>• Resolution of conflicting goals of agriculture and land use
