# Supplementary material for: Scenario Development as a Basis for Formulating a Research Program on Future Agriculture: A Methodological Approach
Source: Ambio. 2013 Jul 9;42(7):823–39. doi: 10.1007/s13280-013-0417-3 (PMC3790135; doi:10.1007/s13280-013-0417-3)
Supplement: Supplementary file 3 — Appendix 1a (PDF 72 kb) [file 13280_2013_417_MOESM3_ESM.pdf]

Appendix 1a Factors and states used to construct the scenarios

GLOBAL PERSPECTIVE

Land-water resource availability, global scenarios (sub-scenarios)	Power relations, global scenarios (sub-scenarios)	Global population 2050	Global economic development	Global climate scenarios (1990–2090)	Global energy supply	Global technological development and distribution	Global food consumption (calories)
GR1 Less of all except land area	GM1 Fragmentation	Slower growth. 8 billion	High north Low south	Min effect, 1–2 °C	Large area Little availability/high price	Rapid development. Even distribution	Animal-based 25 % Plant-based 75 %
GR2 Good availability of all resources	GM2 Unipolar world order, Asia dominates	UN's forecast. 9 billion	Low north High south	Moderate effect, 2–3 °C	Large area Large availability/low price	Rapid development. Uneven distribution	Animal-based 17 % Plant-based 83 %
GR3 Weak biological systems, plentiful inputs	GM3 Unipolar world order, the West dominates	Faster growth. 11 billion	Low north Low south	Max effect, 3–4 °C	Small area Large availability/low price	Slow development. Even distribution	Animal-based 10 % Plant-based 90 %
GR4 Production closer to polar regions	GM4 Multipolar world order		High north High south		Small area Little availability/high price	Slow development. Uneven distribution	
	GM5 Regional protectionism						
	GM6 Adapting mosaic – strong regions that cooperate						

Factors in the sub-scenarios GR1-GR4

Global potential grazing and arable land	Soil fertility Production potential Ecosystem services	Availability of agricultural inputs	Global access to water	Fish and aquaculture
Area as today, situated as today	Increased	Good Low prices	Access to water as today, distributed as today	Availability of wild fish as today
Area as today displaced towards the polar regions	As today	Little High prices	Access to water as today, more unevenly distributed	Less availability of wild fish. Aquaculture makes up the difference
Area as today displaced towards the equator	Decreased		Less access to water than today, distributed as today	Less availability of fish
Increased area			Less access to water than today, more unevenly distributed than today.	

Factors in the sub-scenarios GM1-GM6

Role of large (strong) states	Supranational institutions that affect food supply	Role of private (commercial) actors	Role of non-governmental (political) actors
The USA dominates	Strong globally	Stronger than today	Strong
Multipolar	Functioning globally	As today	As today
China–India dominate	Functioning regionally	Weaker than today	Weaker than today
No strong states	Weaker than today		
