# Supplementary material for: Scenario Development as a Basis for Formulating a Research Program on Future Agriculture: A Methodological Approach
Source: Ambio. 2013 Jul 9;42(7):823–39. doi: 10.1007/s13280-013-0417-3 (PMC3790135; doi:10.1007/s13280-013-0417-3)
Supplement: Supplementary file 4 — Appendix 1b (PDF 71 kb) [file 13280_2013_417_MOESM4_ESM.pdf]

Appendix 1b Factors and states used to construct the scenarios

REGIONAL PERSPECTIVE

Population, regional scenarios (sub-scenarios)	Land-water resource availability, regional scenarios (sub-scenarios))	Agricultural policy	Regional power relations	Economic development in Europe	Regional food consumption (calories)	Animal-based food consumption (protein)
RB1 Continuing urbanisation	RR1 Increased area grazing and arable land	Global trade agreements replace European agreements	Strong supranationalism in Europe	Equalized development in Europe	Animal-based 30 %<br>Plant-based 70 %	Beef & lamb 10 %<br>Milk products 35 %<br>Pork & poultry & eggs 25 %<br>Fish & shellfish 30 %
RB2 Labour migration	RR2 Increased production in the east	Deregulation between Europe and the world	Strong nation states in Europe	Economic balance of power as today	Animal-based 20 %<br>Plant-based 80 %	Beef & lamb 25 %<br>Milk products 25 %<br>Pork & poultry & eggs 40 %<br>Fish & shellfish 10 %
RB3 Urban and rural development	RR3 Weak biological systems, plentiful inputs	Europe a protected market	Weak states. Weak supranationalism in Europe	Stronger in east<br>Weaker in south	Animal-based 10 %<br>Plant-based 90 %	Beef & lamb 15 %<br>Milk products 35 %<br>Pork & poultry & eggs 40 %<br>Fish & shellfish 10 %
RB4 Controlled migration and rural policies	RR4 Decreased production in the south, increased in the north and the east.	National agricultural policies				Beef & lamb 10 %<br>Milk products 20 %<br>Pork & poultry & eggs 50 %<br>Fish & shellfish 20 %
RB5 Uncontrolled migration						Beef & lamb 10 %<br>Milk products 20 %<br>Pork & poultry & eggs 60 %<br>Fish & shellfish 10 %

Factors used in the sub-scenarios RB1-RB5

Housing patterns in the regions	Population size in the regions	Demographics in the regions
High urbanisation<br>Large cities growing	Approximately as today	Even age distribution<br>Obelisk
Urban concentration<br>Small towns growing	Large increase<br>20–50 %	Pyramid with a broad base<br>Many young people
Urban sprawl		
Developed rural areas		

Factors used in the sub-scenarios RR1-RR4

Potential grazing and arable land in the regions	Soil fertility<br>Production potential<br>Ecosystem services	Availability of agricultural inputs	Access to water in the regions	Fish and aquaculture
Area as today, situated as today	Increased	Good<br>Low prices	Access to water as today, distributed as today	Availability of wild fish as today
Area as today displaced towards northern Europe	As today	Little<br>High prices	Access to water as today, more unevenly distributed	Less availability of wild fish. Aquaculture makes up the difference
Area as today displaced towards eastern Europe	Decreased		Less access to water than today, distributed as today	Less availability of fish
Increased area			Less access to water than today, more unevenly distributed than today.	
